# Supplementary material for: Bayesian refinement of protein structures and ensembles against SAXS data using molecular dynamics
Source: PLoS Comput Biol. 2017 Oct 18;13(10):e1005800. doi: 10.1371/journal.pcbi.1005800 (PMC5662244; doi:10.1371/journal.pcbi.1005800)
Supplement: S1 Archive — (BZ2) [file pcbi.1005800.s011.tar.bz2 › gromacs_4.62_bayesian_saxs_refi/share/html/online/g_enemat.html]

g\_enemat


| g\_enemat Main Table of Contents |

**VERSION 4.6  
Sat 19 Jan 2013**

---

### Description

g\_enemat extracts an energy matrix from the energy file (-f).
With -groups a file must be supplied with on each
line a group of atoms to be used. For these groups matrix of
interaction energies will be extracted from the energy file
by looking for energy groups with names corresponding to pairs
of groups of atoms, e.g. if your -groups file contains:  
2  
Protein  
SOL  
then energy groups with names like 'Coul-SR:Protein-SOL' and
'LJ:Protein-SOL' are expected in the energy file (although
g\_enemat is most useful if many groups are analyzed
simultaneously). Matrices for different energy types are written
out separately, as controlled by the
-[no]coul, -[no]coulr, -[no]coul14,
-[no]lj, -[no]lj14,
-[no]bham and -[no]free options.
Finally, the total interaction energy energy per group can be
calculated (-etot).

An approximation of the free energy can be calculated using:
E\_free = E\_0 + kT log(), where '<>'
stands for time-average. A file with reference free energies
can be supplied to calculate the free energy difference
with some reference state. Group names (e.g. residue names)
in the reference file should correspond to the group names
as used in the -groups file, but a appended number
(e.g. residue number) in the -groups will be ignored
in the comparison.

### Files

| option | filename | type | description |
| --- | --- | --- | --- |
| **-f** | ener.edr | Input, Opt. | Energy file |
| **-groups** | groups.dat | Input | Generic data file |
| **-eref** | eref.dat | Input, Opt. | Generic data file |
| **-emat** | emat.xpm | Output | X PixMap compatible matrix file |
| **-etot** | energy.xvg | Output | xvgr/xmgr file |

### Other options

| option | type | default | description |
| --- | --- | --- | --- |
| **-[no]h** | bool | no | Print help info and quit || **-[no]version** | bool | no | Print version info and quit || **-nice** | int | 19 | Set the nicelevel || **-b** | time | 0 | First frame (ps) to read from trajectory || **-e** | time | 0 | Last frame (ps) to read from trajectory || **-dt** | time | 0 | Only use frame when t MOD dt = first time (ps) || **-[no]w** | bool | no | View output .xvg, .xpm, .eps and .pdb files || **-xvg** | enum | xmgrace | xvg plot formatting: xmgrace, xmgr or none || **-[no]sum** | bool | no | Sum the energy terms selected rather than display them all || **-skip** | int | 0 | Skip number of frames between data points || **-[no]mean** | bool | yes | with -groups extracts matrix of mean energies instead of matrix for each timestep || **-nlevels** | int | 20 | number of levels for matrix colors || **-max** | real | 1e+20 | max value for energies || **-min** | real | -1e+20 | min value for energies || **-[no]coulsr** | bool | yes | extract Coulomb SR energies || **-[no]coullr** | bool | no | extract Coulomb LR energies || **-[no]coul14** | bool | no | extract Coulomb 1-4 energies || **-[no]ljsr** | bool | yes | extract Lennard-Jones SR energies || **-[no]ljlr** | bool | no | extract Lennard-Jones LR energies || **-[no]lj14** | bool | no | extract Lennard-Jones 1-4 energies || **-[no]bhamsr** | bool | no | extract Buckingham SR energies || **-[no]bhamlr** | bool | no | extract Buckingham LR energies || **-[no]free** | bool | yes | calculate free energy || **-temp** | real | 300 | reference temperature for free energy calculation |

---

http://www.gromacs.org  
gromacs@gromacs.org
